# Blood Pressure Changes in Relation to Arsenic Exposure in a U.S. Pregnancy Cohort

**DOI:** 10.1289/ehp.1408472

**Published:** 2015-03-20

**Authors:** Shohreh F. Farzan, Yu Chen, Fen Wu, Jieying Jiang, Mengling Liu, Emily Baker, Susan A. Korrick, Margaret R. Karagas

**Affiliations:** 1 Children’s Environmental Health and Disease Prevention Research Center at Dartmouth, Hanover, New Hampshire, USA; 2 Department of Epidemiology, Geisel School of Medicine at Dartmouth, Lebanon, New Hampshire, USA; 3 Department of Population Health, New York University School of Medicine, New York, New York, USA; 4 Department of Obstetrics and Gynecology, Dartmouth Hitchcock Medical Center, Lebanon, New Hampshire, USA; 5 Department of Environmental Health, Harvard School of Public Health, Boston, Massachusetts, USA; 6 Channing Division of Network Medicine, Department of Medicine, Brigham and Women’s Hospital and Harvard Medical School, Boston, Massachusetts, USA

## Abstract

**Background:**

Inorganic arsenic exposure has been related to the risk of increased blood pressure based largely on cross-sectional studies conducted in highly exposed populations. Pregnancy is a period of particular vulnerability to environmental insults. However, little is known about the cardiovascular impacts of arsenic exposure during pregnancy.

**Objectives:**

We evaluated the association between prenatal arsenic exposure and maternal blood pressure over the course of pregnancy in a U.S. population.

**Methods:**

The New Hampshire Birth Cohort Study is an ongoing prospective cohort study in which > 10% of participant household wells exceed the arsenic maximum contaminant level of 10 μg/L established by the U.S. EPA. Total urinary arsenic measured at 24–28 weeks gestation was measured and used as a biomarker of exposure during pregnancy in 514 pregnant women, 18–45 years of age, who used a private well in their household. Outcomes were repeated blood pressure measurements (systolic, diastolic, and pulse pressure) recorded during pregnancy.

**Results:**

Using linear mixed effects models, we estimated that, on average, each 5-μg/L increase in urinary arsenic was associated with a 0.15-mmHg (95% CI: 0.02, 0.29; *p* = 0.022) increase in systolic blood pressure per month and a 0.14-mmHg (95% CI: 0.02, 0.25; *p* = 0.021) increase in pulse pressure per month over the course of pregnancy.

**Conclusions:**

In our U.S. cohort of pregnant women, arsenic exposure was associated with greater increases in blood pressure over the course of pregnancy. These findings may have important implications because even modest increases in blood pressure impact cardiovascular disease risk.

**Citation:**

Farzan SF, Chen Y, Wu F, Jiang J, Liu M, Baker E, Korrick SA, Karagas MR. 2015. Blood pressure changes in relation to arsenic exposure in a U.S. pregnancy cohort. Environ Health Perspect 123:999–1006; http://dx.doi.org/10.1289/ehp.1408472

## Introduction

Millions of individuals are chronically exposed to inorganic arsenic via contaminated water sources and through diet (National Research Council 2014; Navas-Acien and Nachman 2013). In the United States, an estimated 17 million people have been exposed to drinking water sources containing arsenic levels exceeding the maximum contaminant limit of 10 μg/L [U.S. Environmental Protection Agency (EPA) 2000]. Common dietary staples, such as rice and poultry, have been found to contain elevated levels of arsenic that also contribute to an individual’s overall exposure (Cottingham et al. 2013; Davis et al. 2012; Gilbert-Diamond et al. 2011; Nachman et al. 2013; Navas-Acien and Nachman 2013). Arsenic exposure has been associated with adverse health effects, including cancer, diabetes, and cardiovascular disease (National Research Council 2014).

Cardiovascular disease is the leading cause of morbidity and mortality worldwide (World Health Organization 2008), and associations between arsenic and the risk of cardiovascular events have been well documented in highly exposed populations (Chen et al. 2011; Moon et al. 2012, 2013; States et al. 2009). Recent prospective work in the United States observed a relation between low-level arsenic exposure and risk of cardiovascular disease (Moon et al. 2012, 2013). Indeed, a growing body of evidence suggests that arsenic may increase risks of some risk factors for cardiovascular diseases, including high blood pressure (BP), atherosclerosis, and endothelial dysfunction (Chen et al. 2007a, 2007b, 2013; Hsieh et al. 2011; Wang et al. 2007; Wu et al. 2012). However, available evidence on cardiovascular disease risk factors is based on cross-sectional studies, and prospective studies that characterize the magnitudes of longitudinal changes in risk factors related to arsenic exposure are lacking. Moreover, certain populations, such as pregnant women, may be especially susceptible to these adverse effects, but little is known about the cardiovascular effects of arsenic exposure during this time period.

Pregnancy profoundly alters both maternal anatomy and physiology to support fetal development (Cunningham et al. 2010). Pregnancy-induced hemodynamic adaptations and hormonal changes lead to normal fluctuations in gestational BP (Cunningham et al. 2010). However, these changes can act as cardiovascular and metabolic stressors (Yoder et al. 2009), creating a “susceptible window” of risk for development of hypertension from putative triggers, including environmental exposures such as lead and air pollutants (Jedrychowski et al. 2012; Lee et al. 2012; van den Hooven et al. 2011; Yazbeck et al. 2009). Further, high BP during pregnancy can signal a greater risk of later-life maternal cardiovascular disease (Henriques et al. 2014; Irgens et al. 2001; Magnussen et al. 2009; Nisell et al. 1995; Skjaerven et al. 2012; Wilson et al. 2003) and also enhances risk of adverse birth outcomes such as premature labor, placental abruption, and restricted placental blood flow to the fetus, which is related to low birth weight (Allen et al. 2004; Roberts et al. 2005).

In New Hampshire, about 40% of households rely on unregulated private water systems, of which 10–15% contain arsenic levels exceeding the maximum contaminant level (Karagas et al. 2002). As part of the New Hampshire Birth Cohort Study, we sought to investigate whether higher maternal arsenic exposure during pregnancy is related to increases in maternal BP, an early cardiovascular disease risk factor and a complicating factor in pregnancy.

## Methods

*The New Hampshire Birth Cohort.* In January 2009, we began recruiting 18- to 45-year-old pregnant women receiving prenatal care at study clinics, as previously described (Gilbert-Diamond et al. 2011). Women were enrolled at 24–28 weeks gestation if they reported using water from a private well at their residence since their last menstrual period and were not planning to move prior to delivery. Only singleton births are included in the study. All protocols were approved by the Dartmouth College Institutional Review Board. All participants provided written, informed consent upon enrollment.

Participants completed a detailed medical history and lifestyle questionnaire upon enrollment and a follow-up questionnaire at 2 weeks postpartum to provide updated information about changes in key exposures and prenatal complications. After delivery, participants’ medical records were reviewed to abstract pre- and post-delivery health information, including all clinically measured maternal BP levels, diagnoses of gestational diabetes, hypertension, preeclampsia and eclampsia. Other clinical information was recorded to verify self-reported medical and reproductive history. Maternal systolic (SBP) and diastolic (DBP) BP was measured in the study clinics, using either automated or mercury sphygmomanometers, throughout pregnancy and was generally recorded at each prenatal visit.

*Arsenic exposure assessment.* Women provided a spot urine sample upon enrollment, which was collected and stored as previously described (Farzan et al. 2013; Gilbert-Diamond et al. 2011). Urine samples were analyzed for levels of arsenite (iAs^III^), arsenate (iAs^V^), monomethylarsonic acid (MMA), dimethylarsinic acid (DMA), and arsenobetaine by high-performance liquid chromatography (HPLC) inductively coupled plasma mass spectrometry (ICP-MS) at the University of Arizona Hazard Identification Core (Larsen et al. 1993; Le et al. 2000; Wei et al. 2001). Samples that registered below the detection limit (ranging from 0.10 to 0.15 μg/L for individual species; 0.6%, 16.5%, and 37.0% of the study population were below the detection limit for DMA, MMA, and iAs, respectively) were assigned a value equal to the detection limit divided by the square root of 2. Urinary creatinine levels (milligrams per deciliter) were determined using Cayman’s creatinine assay kit, according to the manufacturer’s instructions. Our primary exposure measure was total urinary arsenic at 24–28 weeks gestation, calculated by summing inorganic (iAs = iAs^III^ + iAs^V^) and organic (DMA, MMA) metabolites (Farzan et al. 2013; Gilbert-Diamond et al. 2011). Arsenobetaine, an unmetabolized form of arsenic found in seafood, was excluded because it is considered nontoxic (Tseng 2009). As secondary exposure measures, we examined the absolute values of urinary metabolites (MMA, DMA, and iAs). We also constructed primary (PMI) and secondary methylation indices (SMI) from ratios of MMA to iAs and DMA to MMA in urine, respectively, because these are considered indicators of methylation capacity that may impact individual variability in health effects of arsenic exposure (Chen et al. 2013). Upon enrollment, participants also were given instructions and prepaid mailing materials to collect samples of their home tap water and return the samples to the study office; these samples were analyzed by ICP-MS at the Dartmouth Trace Element Analysis Core, as previously described (Gilbert-Diamond et al. 2011). Maternal toenail samples were collected at 2 weeks postpartum, washed five times by sonication in a solution of Triton X-100 and acetone, followed by deionized water, and then dried before low-pressure microwave digestion. Samples were analyzed for trace elements previously related to BP (i.e., selenium, cadmium, iron, mercury, and lead) (Houston 2007; Kennedy et al. 2012; Wells et al. 2012) using ICP-MS as previously described for arsenic (Davis et al. 2014).

*Statistical analysis.* We confined our analysis to women without a history of hypertension prior to pregnancy with at least two pregnancy BP measurements. Our outcomes of interest were temporal changes in SBP, DBP, and pulse pressure (PP; SBP minus DBP) during pregnancy, which were analyzed as continuous variables with repeated measurements. For each measurement, we calculated the trimester and gestational week, based on the participant’s last menstrual period. We restricted our analysis to measurements taken after 13 weeks gestation due to the low number of measurements recorded before this time. Measurements outside of a reasonable range (i.e., SBP: < 40 or > 250 mmHg, DBP: < 35 or > 180 mmHg) (Lee et al. 2012) were likely incorrectly recorded at time of measurement or incorrectly extracted from the medical record. All values that were excluded were well outside of the physiologically plausible range and were coded as missing (< 1% of measurements, *n* = 9). All other values recorded for these women were within a physiologically reasonable range. There were few cases of diagnosed pregnancy-induced hypertension (*n* = 15) or preeclampsia (*n* = 9) in our study population; thus, it was not possible to analyze these outcomes separately.

We fitted mixed-effect models (Demidenko 2004) of the repeated BP measurements to examine whether maternal urinary total arsenic or arsenic metabolite concentrations influenced SBP, DBP, and PP over the course of pregnancy, as follows:

BP*_ij_* = [β_0_ + β_1_(TIME)*_ij_* + β_2_As_0_*_j_* + β_12_As_0_*_j_*(TIME)*_ij_* + α^T^Z_0_*_j_*] + [μ_0_*_j_* + μ_1_*_j_*(TIME)*_ij_*] + *r_ij_*, [1]

where BP*_ij_* represents BP at time *i* for subject *j*, As_0_*_j_* is urinary arsenic (total, DMA, MMA, or iAs) at baseline (time 0 represents baseline; i.e., the gestational month of each woman’s first BP measurement after 13 weeks gestation) for subject *j*; TIME is gestational month of BP measurement; β_1_ is the coefficient for the association between TIME and BP when arsenic is held constant; β_2_ is the difference in BP for every unit increase in arsenic at baseline; β_12_ is the difference in monthly BP change over pregnancy per unit increase in arsenic (i.e., the estimated effect of arsenic levels on monthly BP change); α^T^ is a row vector of regression coefficients for covariates at baseline (T denotes vector transpose); Z_0_*_j_* is a vector of covariates at baseline. The random intercept μ_0_*_j_* and slope μ_1_*_j_* estimated the within-subject correlation among repeated measurements and between-subject heterogeneity, and *r_ij_* is the error that cannot be accounted for by other covariates and random effects. The terms in the first and second brackets are the fixed and random parts of the model, respectively. We assessed nonlinear trends in the data using the same modeling strategy described above, including model terms to examine the interaction between TIME and categories of arsenic exposure variables (e.g., dummy variables for arsenic tertiles), as well as linearity of the time effect by including an additional interaction term between As_0_*_j_* and TIME^2^. Neither test provided evidence of a nonlinear association (*p* > 0.05). For ease of interpretation, 5 μg/L (~ 1 SD) was used as the unit to report effect estimates for total urinary arsenic and metabolite levels.

Our models were adjusted for available covariates that could potentially influence BP based on *a priori* considerations, including age at enrollment, prepregnancy body mass index (BMI), smoking during pregnancy, marital status, educational attainment, gestational diabetes, parity, and number of BP measurements. As described above, we included the month of gestation during which each BP measurement was obtained in our models. We considered pregnancy BP measurements after 13 weeks gestation (our baseline) in our models because few subjects received BP measurements prior to that time point. Urinary arsenic concentrations were used as a measure of gestational arsenic exposure because urine samples earlier in pregnancy were not available and prior studies suggest that total arsenic concentrations remain relatively stable (Ahmed et al. 2011; Gamble et al. 2006). Because there is some debate as to whether creatinine adjustment is appropriate for urinary arsenic measures, we also tested models with and without urinary creatinine adjustment. We also tested inclusion of arsenobetaine levels as a covariate in our models. We found that neither creatinine nor arsenobetaine adjustment altered our estimates: Results were unchanged with or without creatinine adjustment {i.e., SBP β_12_ 0.15 [95% confidence interval (CI): 0.02, 0.29] with creatinine adjustment}, as well as with or without arsenobetaine adjustment [i.e., SBP β_12_ 0.15 (95% CI: 0.02, 0.29) with arsenobetaine adjustment] (data not shown). For individuals with missing covariate data (Table 1), we used multiple imputation to estimate missing covariate values (Little and Rubin 2002). We examined the missing data patterns; in our models we assumed that the data were missing at random with a monotone structure. We used the regression method within the SAS PROC MI procedure to generate five imputed data sets, then used the PROC MIANALYZE procedure to generate inferences for both the mixed and linear regression models. We also performed sensitivity analyses by excluding participants who smoked during pregnancy or those who developed gestational diabetes to evaluate the impact on our results, because BP may be altered in these groups (Bakker et al. 2010; Bryson et al. 2003; Carpenter 2007; Matkin et al. 1999). We also assessed other exposures from toenail levels as potential confounders. Toenail elements that have been associated with BP in the literature, such as selenium, cadmium, iron, mercury, and lead (Houston 2007; Kennedy et al. 2012; Wells et al. 2012), all had little to very weak correlations with toenail arsenic (*r* < 0.20) (data not shown) and thus were not adjusted for in our analysis.

**Table 1:** Selected characteristics of women enrolled in the New Hampshire Birth Cohort Study (*n *= 514), categorized by tertiles of total urinary arsenic (U‑As) measurements during pregnancy.

Variable	Overall U-As (0.35–288.5 μg/L) *n* = 514	Tertile 1 U-As (0.35–2.54 μg/L)*n* = 171	Tertile 2 U-As(2.54–5.34 μg/L)*n* = 171	Tertile 3 U-As(5.34–288.5 μg/L)*n* = 172
Age at enrollment (years)	31.1 ± 4.9 (18.5–44.6)	30.7 ± 4.9 (19.3–44.4)	31.5 ± 4.9 (18.5–44.6)	31.2 ± 4.9 (19.1–43.4)
Level of education
< 11th grade	4 (0.9)	0 (0)	2 (1.4)	2 (1.4)
High school graduate	43 (9.9)	21 (14.9)	13 (9.0)	9 (6.3)
Junior college, some college, technical school	94 (21.6)	28 (18.8)	30 (20.7)	36 (25.4)
College graduate	173 (39.7)	57 (38.3)	60 (41.8)	56 (39.4)
Postgraduate schooling	122 (28.0)	43 (28.9)	40 (27.6)	39 (27.5)
Missing	78	22	26	30
Relationship status
Married	377 (86.1)	128 (85.3)	130 (89.0)	119 (83.8)
Single	48 (11.0)	19 (12.7)	12 (8.2)	17 (12.0)
Divorced, widowed	13 (3.0)	3 (2.0)	4 (2.7)	6 (4.2)
Missing	76	21	25	30
Prepregnancy BMI (kg/m^2^)	25.1 ± 5.1 (17.6–48.3)	24.5 ± 4.5 (18.0–42.5)	25.2 ± 5.0 (17.6–45.7)	25.7 ± 5.8 (17.6–48.3)
Missing	77	21	25	31
Parity
0	197 (38.5)	70 (41.4)	67 (39.2)	60 (34.9)
1	200 (39.1)	65 (38.5)	64 (37.4)	71 (41.3)
≥ 2	115 (22.5)	34 (20.1)	40 (23.4)	41 (23.8)
Missing	2	2	0	0
Developed gestational hypertension	8 (1.6)	4 (2.3)	1 (0.6)	3 (1.7)
Developed gestational diabetes	36 (7.0)	11 (6.4)	15 (8.8)	10 (5.8)
Smoked during pregnancy	26 (5.8)	10 (2.9)	4 (2.3)	12 (7.0)
Missing	66	18	22	26
Well water arsenic
Mean (μg/L)	4.3 ± 11.0 (0.0–147.7)	2.2 ± 5.9 (0.0–58.0)	3.1 ± 8.2 (0.0–67.5)	7.7 ± 15.9 (0.0–147.7)*
> 10 μg/L MCL	58 (12.5)	10 (5.8)	11 (6.4)	37 (21.5)
Missing	51	15	15	21
MCL, maximum contaminant limit. Data are *n* (%) or mean ± SD (range). Frequencies and means were compared by chi-square or one-way analysis of variance (ANOVA), respectively. *p < 0.001 compared with tertile 1.				

We conducted analyses stratified by PMI or SMI, using the median values (0.89 and 9.66, respectively) as cut points, to assess whether the association between urinary arsenic and BP changes over time differed by these arsenic methylation indices. We also performed analyses stratified by age (below or at/above a median of 30.9 years), history of prior pregnancy (nulliparous or parous), prepregnancy BMI (< 25 or ≥ 25 kg/m^2^).

**Figure 1:**
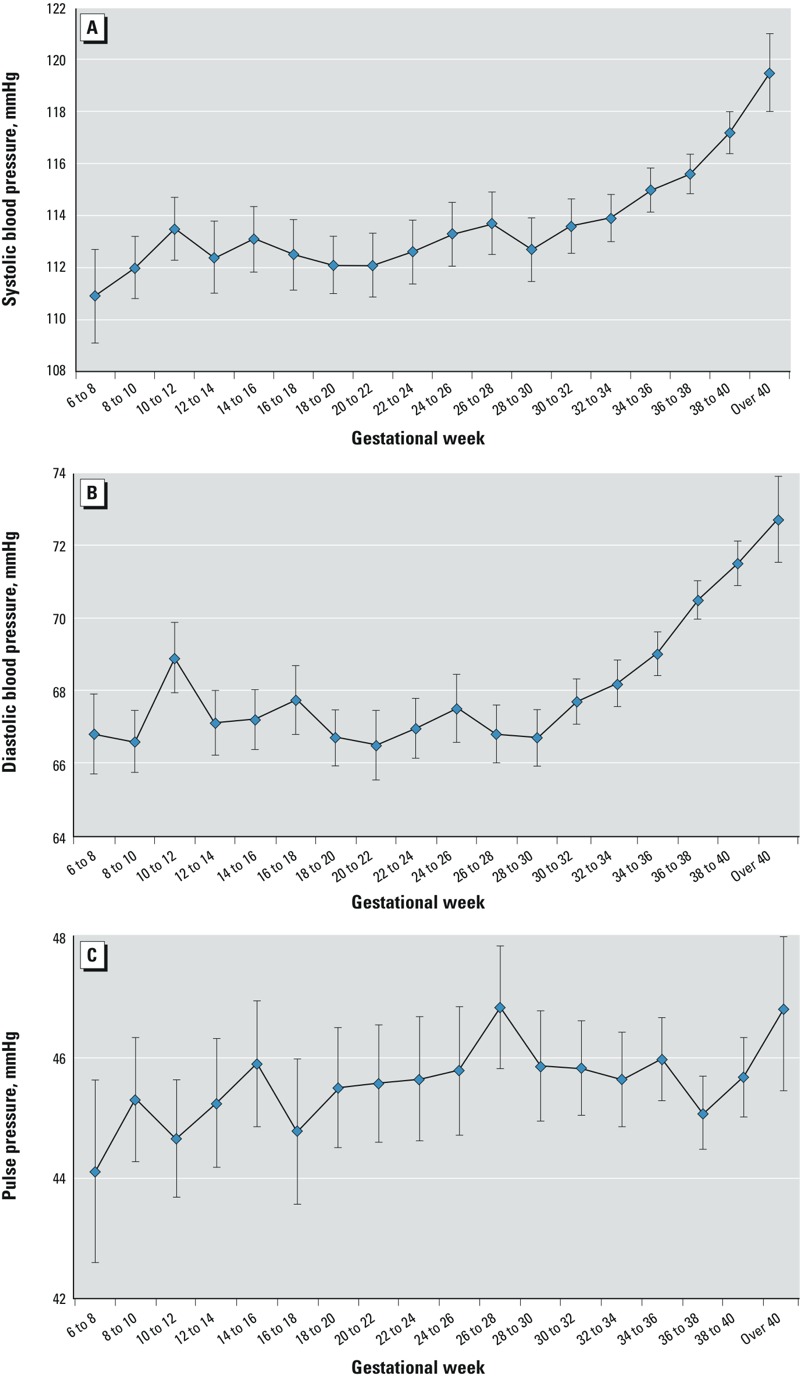
Blood pressure measurements over pregnancy by gestational week. For each 2-week period, all systolic blood pressure (*A*), diastolic blood pressure (*B*), or pulse pressure (*C*) measurements during that time were averaged individually for each woman and then averaged across all women and plotted. Error bars represent the 95% CIs. Measurements prior to 6 weeks of gestation were excluded due to few available measurements.

Because BP increases over the latter part of pregnancy (Cunningham et al. 2010; Miller et al. 2007; Thompson et al. 2007), we further examined whether women with higher urinary arsenic had higher BP at the end of pregnancy, using linear regression models with the outcome, respectively defined as the average of the last three BP measurements (SBP, DBP, PP), adjusting for the same covariate variables. The equation generated from the multivariable linear regression model was also used to graphically represent the relationship between maternal urinary arsenic and SBP at the end of pregnancy, when all covariates are set equal to the median values (Figure 1). In all analyses, *p*-values < 0.05 were considered significant. All analyses were performed using SAS 9.3 (SAS Institute Inc.).

## Results

As of 30 October 2013, 620 participants had available urinary arsenic measurements and, of these, 590 had available medical record review data. As an *a priori* selection requirement, we required women to have at least two BP measurements taken during pregnancy; however, all 527 women in this sample had a minimum of four measurements. An additional 13 women with a history of hypertension were excluded, resulting in a final sample size of 514. This subset was similar to the overall cohort (*n* = 620) with respect to demographic and lifestyle variables (data not shown). Women in this sample had urinary arsenic concentrations ranging from 0.35 to 288.5 μg/L, which was very similar to the range observed in the overall cohort (0.08–288.5 μg/L). Of the subset of participants included in these analyses, 472 had provided water samples, 463 of which had been analyzed at the time of this study and had a mean arsenic level of 4.3 μg/L (range, 0–147.7 μg/L). Nearly 1 in every 8 households (58 of 463 available samples; 12.5%) tested in this subsample had water arsenic levels > 10 μg/L. In our study group, well water arsenic levels were significantly correlated with urinary arsenic measurements (*r* = 0.40, *p* < 0.001). No differences in the descriptive variables were observed across urinary arsenic tertiles by chi-square or one-way analysis of variance (ANOVA) tests, except for water arsenic levels, in which the third tertile was higher than the first tertile (Table 1).

The 514 women in this subsample of the cohort contributed a total of 6,675 SBP and 6,671 DBP measurements (5,773 SBP and 5,769 DBP after gestational week 13). On average, 13 (range, 4–24) BP measurements were recorded per participant during pregnancy, with more than half occurring in the last trimester. Both SBP and DBP increased during pregnancy (Figure 1; see also Supplemental Material, Table S1), with the highest averages for both SBP and DBP occurring in the third trimester (SBP trimester mean ± SD: first, 112.9 ± 9.8; second, 112.5 ± 8.2; third, 115.3 ± 8.4; DBP trimester mean ± SD: first, 68.4 ± 7.7; second, 67.0 ± 6.0; third, 69.4 ± 6.2). PP also appeared to increase over the course of pregnancy, with somewhat more variability (Figure 1; see also Supplemental Material, Table S1).

We observed no association between urinary arsenic and differences in DBP change over pregnancy. Arsenic exposure was related to greater monthly increases in SBP and PP change over the course of pregnancy (Table 2). Based on our model, each 5-μg/L increase in urinary arsenic was associated with a 0.15-mmHg greater monthly increase in SBP (95% CI: 0.02, 0.29; *p* = 0.022) and a 0.14-mmHg greater monthly increase in PP (95% CI: 0.02, 0.25; *p* = 0.021) over the course of pregnancy (Table 2). In sensitivity analyses, excluding smokers or individuals with gestational diabetes did not appreciably alter any of these findings (data not shown). The metabolites MMA, DMA, and iAs were all positively associated with greater increases in PP over the course of pregnancy [MMA β_12_, 1.54 (95% CI: 0.16, 2.92); DMA β_12_, 0.15 (95% CI: 0.02, 0.29); iAs β_12_, 1.18 (95% CI: –0.01, 2.38)] (Table 2). Higher levels of DMA were also associated with greater increases in SBP [β_12_, 0.18 (95% CI: 0.02, 0.33)] over the course of pregnancy.

**Table 2:** Relation between pregnancy urinary arsenic and changes in blood pressure (mmHg) per month over pregnancy among 514 women in the New Hampshire Birth Cohort Study.

As exposure measure (per 5 μg/L)	No. of BP measurements	SBP		DBP		PP	
		β_12_ (95% CI)^*a*^	*p*‑Value^*b*^	β_12_ (95% CI)^*a*^	*p*‑Value^*b*^	β_12_ (95% CI)^*a*^	*p*‑Value^*b*^
Total As	5,032	0.15 (0.02, 0.29)	0.022	0.02 (–0.08, 0.12)	0.73	0.14 (0.02, 0.25)	0.021
MMA	5,016	1.28 (–0.27, 2.83)	0.11	–0.25 (–1.45, 0.96)	0.69	1.54 (0.16, 2.92)	0.028
DMA	5,032	0.18 (0.02, 0.33)	0.022	0.03 (–0.09, 0.14)	0.67	0.15 (0.02, 0.29)	0.027
iAs	5,031	1.11 (–0.23, 2.44)	0.10	–0.01 (–1.04, 1.03)	0.98	1.18 (–0.01, 2.38)	0.052
Abbreviations: BP, blood pressure; DBP, diastolic blood pressure; PP, pulse pressure; SBP, systolic blood pressure. ^***a***^Coefficient in relation to interaction between a 5-μg/L increase in total urinary arsenic, MMA, DMA, or iAs and each month of gestation; adjusted for age at enrollment, prepregnancy BMI, educational level, marital status, maternal smoking, parity, gestational diabetes, and number of blood pressure measurements per participant. ^***b***^*p*-Values for β_12_ effect estimates.							

Women with higher PMI had greater average increases in both SBP and PP over the course of pregnancy compared with those with lower PMI, although the differences from women with lower PMI were not significant [SBP β_12_, 0.23 (95% CI: 0.07, 0.39) vs. β_12_, 0.06 (95% CI: –0.17, 0.29; *p*-interaction = 0.21); PP β_12_, 0.18 (95% CI: 0.03, 0.34) vs. β_12_, 0.10 (95% CI: –0.10, 0.29; *p*-interaction = 0.47)] (Table 3). Similarly, those with higher SMI appeared to have greater increases in SBP [high SMI β_12_, 0.25 (95% CI: 0.07, 0.42) vs. low SMI β_12_, 0.05 (95% CI: –0.15, 0.26; *p*-interaction = 0.17)] over the course of pregnancy, although the test for interaction was not significant. No effect modification by SMI was observed for PP (high SMI β_12_, 0.16 (95% CI: 0.01, 0.31) vs. low SMI β_12_, 0.12 (95% CI: –0.06, 0.31; *p*-interaction = 0.74)]. In analyses stratified by potential effect modifiers, including prepregnancy BMI, age, and parity, we observe no statistically significant associations between total urinary arsenic and longitudinal changes in BP (data not shown). We also examined whether individuals with missing data affected the outcomes and found that, when individuals with missing covariate information (*n* = 78) were excluded, the estimates were nearly unchanged [i.e., SBP β_12_, 0.15 (95% CI: 0.02, 0.28)] (data not shown).

**Table 3:** Relation between pregnancy total urinary arsenic and changes in blood pressure (mmHg) over pregnancy among 514 women in the New Hampshire Birth Cohort Study, stratified by methylation indices.

Methylation index	No. of BP measurements	SBP		DBP		PP	
		β_12_ (95% CI)^*a*^	*p*‑Value^*b*^	β_12_ (95% CI)^*a*^	*p*‑Value^*b*^	β_12_ (95% CI)^*a*^	*p*‑Value^*b*^
PMI
Low	2,535	0.06 (–0.17, 0.29)	0.60	–0.02 (–0.19, 0.14)	0.76	0.10 (–0.10, 0.29)	0.33
High	2,475	0.23 (0.07, 0.39)	0.004	0.05 (–0.09, 0.19)	0.51	0.18 (0.03, 0.34)	0.021
*p* for interaction^*c*^			0.21		0.52		0.47
SMI	
Low	2,464	0.05 (–0.15, 0.26)	0.61	–0.06 (–0.22, 0.10)	0.47	0.12 (–0.06, 0.31)	0.19
High	2,546	0.25 (0.07, 0.42)	0.005	0.09 (–0.05, 0.22)	0.22	0.16 (0.01, 0.31)	0.040
*p* for interaction^*c*^			0.17		0.17		0.74
Abbreviations: BP, blood pressure; DBP, diastolic blood pressure; PMI, primary methylation index (MMA/iAs); PP, pulse pressure; SBP, systolic blood pressure; SMI, secondary methylation index (DMA/MMA). ^***a***^Coefficient in relation to interaction between a 5-μg/L increase in total urinary arsenic and each month of gestation; adjusted for age at enrollment, prepregnancy BMI, educational level, marital status, maternal smoking, parity, gestational diabetes, and number of blood pressure measurements per participant. ^***b***^*p*-Values for β_12_ effect estimates. ^***c***^*p* for interaction, based on two-tailed tests of significance.							

When we conducted simple linear regression models, based on the average of the last three BP measurements, each 5-μg/L increase in urinary arsenic was associated with a 0.78-mmHg (95% CI: 0.05, 1.51; *p* = 0.035) higher SBP. Again, total urinary arsenic was unrelated to DBP [β = 0.34 (95% CI: –0.20, 0.89; *p* = 0.22)]. Likewise, as in the longitudinal analysis, PP was positively associated with urinary arsenic, but with wide confidence intervals [β = 0.44 (95% CI: –0.10, 0.97; *p* = 0.11)]. Graphical representation of the linear regression model depicts an increase in the average of the last three SBP measurements in relation to urinary arsenic level (see Supplemental Material, Figure S1).

## Discussion

To our knowledge, our study is the first prospective study to examine the association between arsenic and BP in the context of pregnancy and among the few studies on the cardiovascular effects of arsenic exposure in the United States. Because pregnancy is a vulnerable window of susceptibility to adverse BP changes, by focusing on a cohort of pregnant women we found that higher levels of urinary arsenic during pregnancy prospectively related to greater increases in SBP and PP over the course of pregnancy.

Arsenic has been associated with a range of cardiovascular outcomes in populations with appreciable levels of chronic exposure, such as in Bangladesh and Taiwan, including increased risks of fatal and nonfatal cardiovascular disease, as well as intermediary factors, such as increased carotid intima-media thickness and metabolic syndrome (Chen CJ et al. 1995; Chen Y et al. 2007a, 2011, 2013; Kwok et al. 2007; Wang et al. 2007; Wu et al. 1989). Arsenic has been associated with hypertension in a number of cross-sectional studies, from which a meta-analysis derived a pooled odds ratio for hypertension of 1.27 (95% CI: 1.09, 1.47) for high versus low arsenic exposure (Abhyankar et al. 2012). Although potential causal mechanisms for the association between arsenic and BP increase during pregnancy have not yet been explored, many of the mechanisms hypothesized to explain associations with other cardiovascular outcomes could be involved. Arsenic exposure has been related to increased plasma markers of inflammation and endothelial damage (Burgess et al. 2013; Chen et al. 2007b; Wu et al. 2012), suggesting that arsenic may act in part by promoting endothelial dysfunction, pathologic vascular remodeling, and atherosclerosis. Thus, while speculative, arsenic exposure could impact the pregnancy-related hemodynamic adaptations that increase blood volume and maintain placental perfusion, which is critical to fetal nutrient and oxygen supply.

BP normally increases toward the latter part of pregnancy, with increases in SBP generally tending to be somewhat more pronounced than those in DBP (Cunningham et al. 2010; Miller et al. 2007; Thompson et al. 2007). A prospective study of longitudinal BP during pregnancy reported average increases of about 3.7 mmHg and 2.2 mmHg between the first and third trimesters for SBP and DBP, respectively (Miller et al. 2007). Abnormal increases pose a serious risk of complications during pregnancy such as preterm birth, low birth weight, fetal growth restriction, and perinatal mortality (Ray et al. 2001; Xiong and Fraser 2004; Zhang et al. 2007) and the deleterious effects of gestational hypertension (defined as new onset of SBP > 140 mmHg and/or DBP > 90 mmHg in second trimester) are well known. However, elevations in BP that do not exceed the upper threshold of the normal range (SBP < 140 mmHg and DBP < 90 mmHg) may also pose risks to the mother and child. For nonpregnant adults, the risk of cardiovascular disease increases linearly as BP increases, even within the normotensive range (Vasan et al. 2001; Williams et al. 2008). A few studies have examined BP as a continuous measure and found that higher BP, even within the normotensive range, also may impact birth weight and intrauterine growth restriction (Churchill et al. 1997; Fukushima et al. 2012). It is possible that elevated BP, albeit within the clinically normal range, alters uterine and placental perfusion, and impacts fetal growth. Our results suggest that there were greater increases in SBP and PP over pregnancy associated with higher arsenic exposure, leading to greater relative differences at the end of pregnancy. However, the clinical significance of greater increases in BP remains to be explored, and more studies utilizing continuous BP outcome measures are needed to examine the relation between BP elevations within the normal range and health risks.

Pregnancy itself is a cardiovascular stressor. In a rodent study, normal, healthy pregnancies were found to induce long-term alterations in cardiovascular and renal function that were absent in nonparous females (Gallo et al. 2012). Pregnancy-induced hypertension has been associated with increased later life risk of chronic hypertension, endothelial dysfunction, and kidney disease (Henriques et al. 2014; Nisell et al. 1995; Vikse et al. 2008; Wang et al. 2013; Wilson et al. 2003). According to a recent study, women with a history of a hypertensive pregnancy had nearly 60% greater odds of peripheral artery disease compared with those with normotensive histories, even decades after pregnancy (Weissgerber et al. 2013). Additional longitudinal studies are needed to determine whether BP changes during pregnancy, such as those observed in relation to arsenic exposure in our cohort, lead to long-term health consequences for mother and child.

In our study, we found that each 5 μg/L urinary arsenic was associated with an average SBP increase of 0.15 mmHg per month and a 0.78-mmHg (95% CI: 0.05, 1.51; *p* = 0.035) higher SBP. Although we are unaware of any previous studies of arsenic and BP during pregnancy, recent studies have found that exposure to other environmental contaminants may affect BP during pregnancy with similar magnitudes of effects as observed in our study. Several studies have observed associations between particulate air pollution and increased BP in pregnant women (Lee et al. 2012; van den Hooven et al. 2011), including a prospective study of 431 pregnant women that found third-trimester SBP increased linearly with second-trimester exposure to air particulates (Jedrychowski et al. 2012). The Generation R Study found that a 10-μg/m^3^ increase in PM_10_ exposure was associated with greater increases in SBP over the second and third trimesters: 1.11 (95% CI: 0.43, 1.79) and 2.11 (95% CI: 1.34, 2.89) mmHg, respectively (van den Hooven et al. 2011). A recent U.S. cohort study of air pollution on BP changes over the course of pregnancy found that interquartile increases in PM_10_ (particulate matter ≤ 10 μm in aerodynamic diameter) and ozone exposure in the first trimester were associated with average SBP increases of 1.9 mmHg (95% CI: 0.84, 2.93) and 1.8 mmHg (95% CI: 1.05, 4.63), respectively, an association that was more pronounced in nonsmoking mothers (Lee et al. 2012). In addition, a cohort study of 1,017 pregnant women in France found an association between midpregnancy blood lead levels and increased risk of pregnancy-induced hypertension in the second and third trimesters (Yazbeck et al. 2009). Although studies of the impacts of environmental toxicants on cardiovascular effects during pregnancy are increasing, more studies are needed to assess the vulnerable times of exposure, as well as the effects of toxicants known to increase cardiovascular disease in nonpregnant adults, including arsenic.

Ingested inorganic arsenic is primarily metabolized via methylation, first to MMA, then to DMA. Arsenic metabolism varies greatly between individuals, and higher MMA proportions are indicative of inefficient methylation (Buchet et al. 1981; Vahter 1999). MMA, thought to be a more toxic metabolite, has been linked to adverse health effects, including cardiovascular effects (Chen et al. 2013; Huang et al. 2009). Because previous work from more highly exposed individuals has indicated that higher PMI may be associated with greater health risks (Chen et al. 2013), one might expect to see stronger effects only in those with high PMI, which could indicate inefficient arsenic metabolism, as opposed to high SMI, which may indicate more efficient methylation and therefore arsenic excretion. However, we observed associations between urinary arsenic and BP both among those with higher PMI or higher SMI, although differences may have occurred by chance. In populations with lower overall levels of exposure, one might predict that the majority of ingested arsenic, once methylated to MMA, would be more easily methylated to DMA. This prediction is consistent with our observations, as well as with those in other U.S. populations, including recent results from the Strong Heart Study, which indicated that higher DMA proportions were linked to cardiovascular disease incidence and mortality, raising the possibility for a role of higher SMI in cardiovascular risk in populations with low arsenic exposure levels (Moon et al. 2013). A low SMI may be a susceptibility factor in more highly exposed populations, such as in Bangladesh. Further, the pregnancy-related health outcomes related to high SMI (i.e., high DMA levels) are less well understood. It is possible that women with altered arsenic metabolism may be more susceptible to arsenic’s cardiovascular effects and more likely to experience increases in BP during pregnancy. Interestingly, in late pregnancy, a greater proportion of arsenic is excreted as MMA (Concha et al. 1998; Hopenhayn et al. 2003), possibly representing a detoxification mechanism. Although this pregnancy-related alteration in metabolism is not well understood, it is possible that this mechanism may in part account for the observed association between increased BP in association with both PMI and SMI. Further study of the effect modification by arsenic metabolites is warranted, particularly at the lower levels of arsenic exposure found in U.S. populations.

Urinary arsenic is considered to be a reliable short-term measure of arsenic exposure that appears to remain relatively consistent in adults, even during pregnancy (Ahmed et al. 2011; Gamble et al. 2006). In the present study, we collected urine samples over a narrow gestational time frame, during which concentrations were previously found not to vary (Gilbert-Diamond et al. 2011). To examine the trajectory of BP over pregnancy, we used measurements beginning at 13 weeks gestation; thus, some measurements were taken prior to urine sampling. However, prior studies suggest that total urinary arsenic levels remain relatively constant over pregnancy (Ahmed et al. 2011). However, our single exposure measurement may not be representative of typical exposure levels for all of the women in our study sample, and there may be variability in arsenic exposure levels that we were unable to account for in this study. Further, the study by Gamble et al. (2006) was performed in adults, and urinary arsenic stability may vary between nonpregnant and pregnant adults. Although we did not collect multiple urine samples from participants, we collected maternal toenail samples prior to delivery, which approximately represent the previous 6–9 months of exposure. Among 334 women in our study with both prenatal urinary and toenail arsenic measurements, toenail arsenic was positively correlated with urinary arsenic measurements (*r* = 0.33, *p* < 0.001; data not shown). Moreover, use of urine as an arsenic biomarker allows us to account for exposure from other sources, such as diet. Nearly 1 in 8 individuals (12.5%) in this sample had water arsenic levels that exceeded the U.S. EPA maximum contaminant limit of 10 μg/L, which likely represents the primary source of arsenic exposure among these individuals. Further, work from our study area of New Hampshire has found that a variety of foods, including rice, can also significantly increase an individual’s arsenic exposure (Cottingham et al. 2013; Gilbert-Diamond et al. 2011).

Our study has some potential limitations. First, we used measurements of BP at prenatal care visits, obtained from medical records. These measurements reflect the types of measurements and patterns that are obtained in routine clinical settings; although standard medical procedures were used, differences in staff and instrumentation may have introduced random variability into our measurements. In addition, BP can fluctuate acutely in relation to anxiety, recent exertion, and caffeine consumption, contributing to measurement error. Although we were not able to account for these factors in our models, we would not expect instrumentation to be related to exposure status and error in the precision of measurement techniques that would likely bias our estimates toward the null. We also were unable to account for dietary factors (i.e., high sodium consumption, nutrient levels) that have the potential to impact BP levels, and due to sample size, we may have been limited in our ability to examine the impact of effect modifiers, such as age or BMI. Our study population of mothers tended to be well-educated and primarily white, which may underrepresent different racial or socioeconomic groups that are at higher risk of gestational hypertension. Nonetheless, internal validity of the study is strengthened by the fact that we have multiple measurements for each woman over the course of pregnancy, detailed medical history, and sociodemographic information from our participants to include in our models. However, some women in our study were missing covariate information. We used multiple imputation methods to impute missing data, and we cannot rule out the possibility that data were not missing completely at random. Further, our choice of mixed models helps to account for random variability. Longitudinal data analysis provides a sensitive tool for characterizing health outcomes that change gradually, such as BP, and repeated measures can be a powerful way to identify small changes that can have a large impact at the population level (Farrington 1991). BP has a strong, continuous positive association with cardiovascular disease (Law et al. 2003; MacMahon et al. 1990; Sagie et al. 1993), and as SBP increases above 115 mmHg, the risk of cardiovascular disease rises continuously (Vasan et al. 2001; Williams et al. 2008). Therefore, the changes observed here have the potential to impact maternal cardiovascular risks (Law et al. 2003).

It is becoming increasingly evident that pregnant women and developing fetuses are particularly vulnerable to environmental insults. Inorganic arsenic consumed in both drinking water and diet may contribute to overall arsenic burden in U.S. pregnant women. Although the adverse cardiovascular effects of arsenic have been investigated in adults, to our knowledge, our study is among the first to examine these impacts during pregnancy. As cardiovascular morbidity and mortality rise worldwide, the potential risk of later-life cardiovascular diseases in mothers and children who are exposed to arsenic during pregnancy makes this a critical area of investigation.

## Supplemental Material

(351 KB) PDF
