# Supplementary material for: Blood Pressure Changes in Relation to Arsenic Exposure in a U.S. Pregnancy Cohort
Source: Environ Health Perspect. 2015 Mar 20;123(10):999–1006. doi: 10.1289/ehp.1408472 (PMC4590746; doi:10.1289/ehp.1408472)
Supplement: (351 KB) PDF [file ehp.1408472.s001.acco.pdf]

Environ Health Perspect
doi: 10.1289/ehp.1408472

Note to Readers: *EHP* strives to ensure that all journal content is accessible to all readers. However, some figures and Supplemental Material published in *EHP* articles may not conform to 508 standards due to the complexity of the information being presented. If you need assistance accessing journal content, please contact ehp508@niehs.nih.gov. Our staff will work with you to assess and meet your accessibility needs within 3 working days.

Supplemental Material

Blood Pressure Changes in Relation to Arsenic Exposure in a U.S. Pregnancy Cohort

Shohreh F. Farzan, Yu Chen, Fen Wu, Jieying Jiang, Mengling Liu, Emily Baker, Susan A. Korrick, and Margaret R. Karagas

Table of Contents

Table S1. Mean blood pressure by gestational week.

Figure S1. Graphical representation of relationship between maternal urinary arsenic at gestational week 24-28 (x-axis) and systolic blood pressure at the end of pregnancy (average of last three measurements during pregnancy) (y-axis), as estimated by linear regression. The solid line represents the linear regression equation, when covariates are set equal to the median values in the regression equation (age at enrollment: 30.96 years, pre-pregnancy BMI: 24.02 kg/m^2, educational level (completed college), marital status (married), maternal smoking (no), parity (1 child), and gestational diabetes (no)); the dotted lines represent the 95% confidence interval.

Table S1. Mean blood pressure by gestational week.

Gestational Week	Women with measurements (N)	Mean ± SD			
		Measurements per woman	SBP, mmHg	DBP, mmHg	PP, mmHg
Less than 4	4	1 ± 0	115.0 ± 3.5	71 ± 6.6	42.8 ± 7.9
4-6	13	1.1 ± 0.5	116.0 ± 7.5	70.4 ± 7.3	46.3 ± 9.6
6-8	110	1.1 ± 0.3	110.9 ± 10.2	67.1 ± 6.1	44.1 ± 8.5
8-10	227	1.1 ± 0.3	112.0 ± 8.7	66.6 ± 6.9	45.3 ± 8.2
10-12	293	1.1 ± 0.3	113.5 ± 11.2	68.9 ± 8.7	44.7 ± 8.7
12-14	234	1.1 ± 0.2	112.8 ± 10.9	67.3 ± 7.5	45.3 ± 8.6
14-16	289	1.0 ± 0.2	113.1 ± 11.5	67.2 ± 7.6	45.9 ± 9.2
16-18	198	1.0 ± 0.2	112.4 ± 10.2	67.7 ± 6.8	44.8 ± 8.8
18-20	331	1.0 ± 0.2	111.7 ± 10.2	66.3 ± 7.8	45.5 ± 9.3
20-22	215	1.0 ± 0.2	112.1 ± 8.9	66.6 ± 7.2	45.6 ± 7.5
22-24	285	1.0 ± 0.2	112.4 ± 10.5	66.8 ± 7.2	45.6 ± 9.0
24-26	242	1.1 ± 0.3	113.3 ± 10.0	67.4 ± 7.9	45.8 ± 8.7
26-28	313	1.1 ± 0.3	113.8 ± 10.6	66.9 ± 7.5	46.8 ± 9.5
28-30	344	1.1 ± 0.2	112.7 ± 9.8	66.6 ± 8.0	45.9 ± 8.8
30-32	439	1.4 ± 0.4	113.4 ± 10.8	67.6 ± 7.5	45.8 ± 8.9
32-34	411	1.2 ± 0.6	113.9 ± 10.1	68.0 ± 7.7	45.6 ± 9.0
34-36	451	1.4 ± 0.7	114.4 ± 9.8	68.6 ± 7.3	46.0 ± 8.8
36-38	466	1.8 ± 0.7	115.4 ± 10.1	70.5 ± 7.6	45.1 ± 8.7
38-40	395	1.7 ± 0.7	117.0 ± 9.4	71.3 ± 7.5	45.7 ± 8.8
Over 40	152	1.7 ± 1.2	119.1 ± 11.6	72.7 ± 7.8	46.8 ± 11.1

SBP: systolic blood pressure; DBP: diastolic blood pressure; PP: pulse pressure.

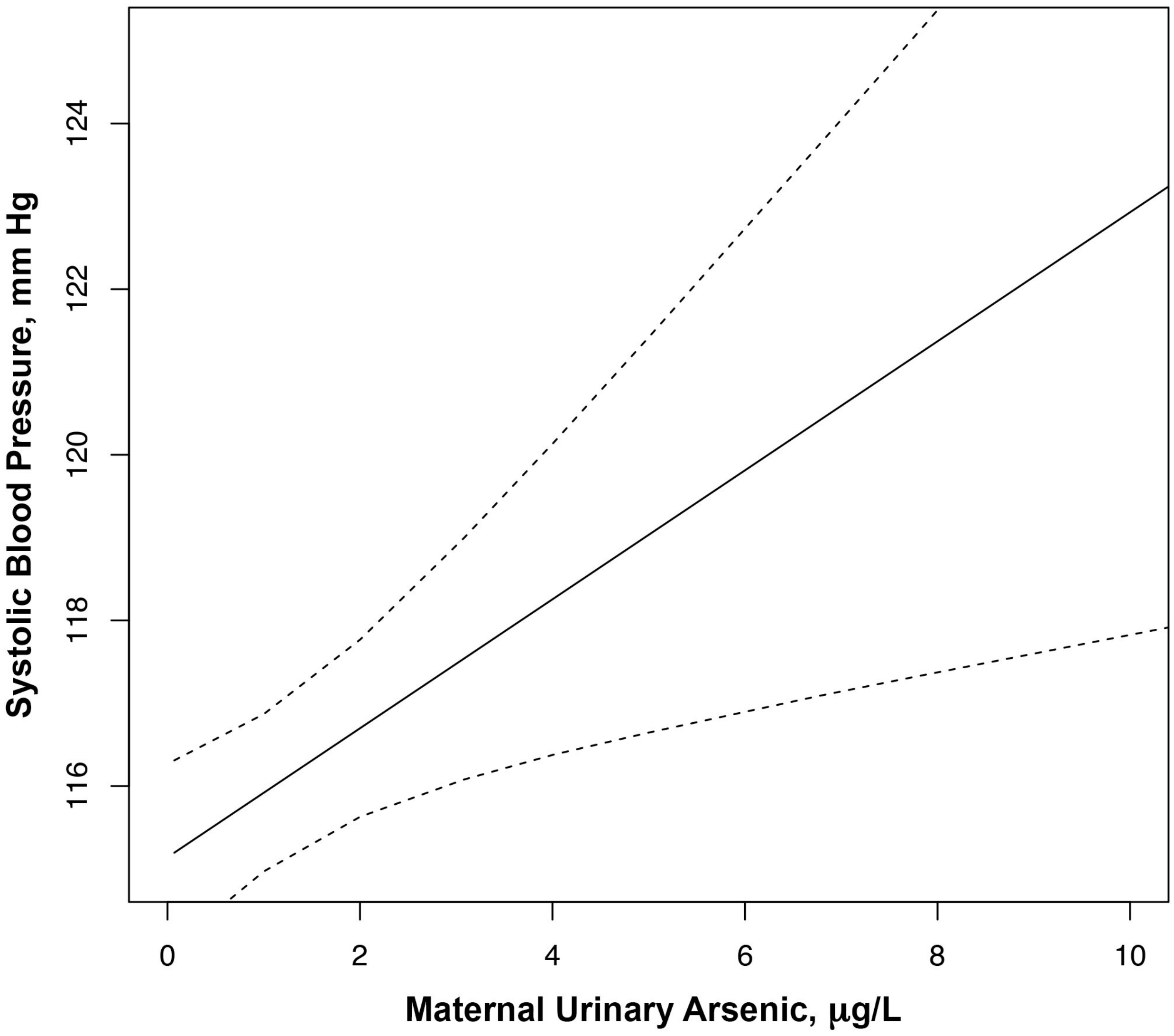

Figure S1. Graphical representation of relationship between maternal urinary arsenic at gestational week 24-28 (x-axis) and systolic blood pressure at the end of pregnancy (average of last three measurements during pregnancy) (y-axis), as estimated by linear regression. The solid line represents the linear regression equation, when covariates are set equal to the median values in the regression equation (age at enrollment: 30.96 years, pre-pregnancy BMI: 24.02 kg/m^2, educational level (completed college), marital status (married), maternal smoking (no), parity (1 child), and gestational diabetes (no)); the dotted lines represent the 95% confidence interval.
